# Therapeutic Effect of Costunolide in Autoimmune Hepatitis: Network Pharmacology and Experimental Validation

**DOI:** 10.3390/ph16020316

**Published:** 2023-02-17

**Authors:** Zheng Huang, Shangshu Nie, Shuhui Wang, Han Wang, Jin Gong, Wei Yan, Dean Tian, Mei Liu

**Affiliations:** Department of Gastroenterology, Tongji Hospital of Tongji Medical College, Huazhong University of Science and Technology, Wuhan 430000, China

**Keywords:** costunolide, autoimmune hepatitis, network pharmacology, immune-mediated liver injury, PI3K-AKT, SRC, IGF1R

## Abstract

Novel treatments for autoimmune hepatitis (AIH) are highly demanded due to the limitations of existing therapeutic agents. Costunolide is a promising candidate due to its anti-inflammatory and hepatoprotective function, but its effect in AIH remains obscure. In this study, we integrated network pharmacology and experimental validation to reveal the effect and mechanism of costunolide in AIH. A total of 73 common targets of costunolide and AIH were obtained from databases. Pathway enrichment analysis indicated that PI3K-AKT pathway was the core pathway of costunolide in AIH. Protein–protein interaction network analysis and molecular docking revealed that SRC and IGF1R might play critical roles. In two murine AIH models, costunolide significantly attenuated liver injury, inflammation, and fibrosis reflected by the liver gross appearance, serum transaminases, necrosis area, spleen index, immune cell infiltration, and collagen deposition. Western blot and immunohistochemistry confirmed that phosphorylated AKT, SRC, and IGF1R were upregulated in AIH models, and costunolide administration could inhibit the phosphorylation of these proteins. In summary, costunolide significantly ameliorates murine AIH. The therapeutic effect might work by suppressing the activation of PI3K-AKT pathway and inhibiting the phosphorylation of SRC and IGF1R. Our research reveals the potent therapeutic effect of costunolide in AIH and the potential role of SRC and IGF1R in AIH for the first time, which may further contribute to the novel drug development for AIH and other autoimmune diseases.

## 1. Introduction

Autoimmune hepatitis (AIH) is a chronic inflammatory liver disease due to immune-mediated destruction of hepatocytes. The etiology of AIH involves the interaction of both genetic background and environmental triggers, which is not entirely understood. Its pathophysiological processes are believed to involve specific genetic traits, molecular mimicry, impaired immunoregulatory mechanisms, etc. [1]. AIH is characterized biochemically by the elevation of serum transaminase, serologically by the presence of autoantibodies and elevated immunoglobulin G (IgG) levels, and histologically by interface hepatitis [2]. It occurs worldwide in all ethnicities, affecting all ages with distinct female preponderance [2]. Although most patients respond well to the standard immunosuppressive therapy of steroids and azathioprine, insufficient response and intolerable side effects occur in 10–20% of patients [3]. Long-term or uncontrolled AIH may lead to progressive fibrosis, cirrhosis, liver failure, and even hepatocellular carcinoma [4]. Therefore, identification of novel therapeutic drugs and clarifying the underlying mechanism is of great significance for the treatment of AIH. 

Traditional Chinese medicine (TCM) and natural products may provide some new options for the drug discovery of AIH [5]. Costunolide (COS) is a well-known sesquiterpene lactone in the germacranolides series isolated from Aucklandiae Radix (Mu Xiang in Chinese). Numerous preclinical studies have indicated that costunolide possesses antioxidative, anti-inflammatory, antiallergic, anticancer, and antidiabetic properties [6]. Remarkably, costunolide exerts a powerful anti-inflammation effect in multiple immune-related diseases such as carrageenan-induced paw edema and lung inflammation, ethanol-induced gastric ulcer, lipoteichoic acid-induced acute lung injury, and dextran sulfate sodium (DSS)-induced murine colitis [7,8,9,10,11,12]. Meanwhile, costunolide exhibits a hepatoprotective effect against lipopolysaccharide and D-galactosamine-induced acute liver injury and alcoholic liver injury [13,14]. It also ameliorates liver fibrosis in vitro and in vivo [15]. However, the effect of costunolide in AIH remains obscure. 

**Figure 1:**
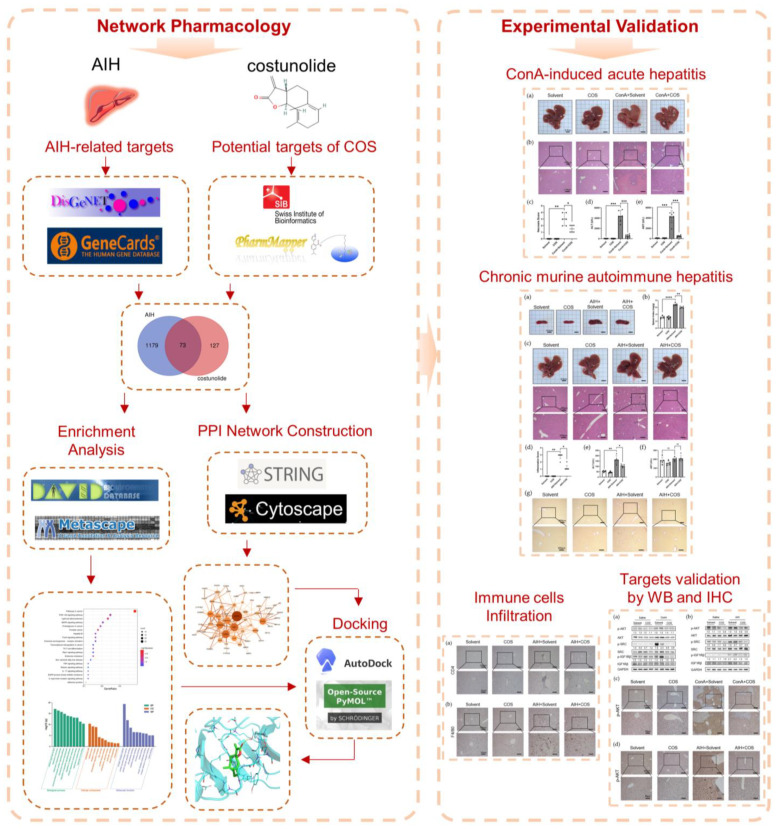
Flow chart of research. Detailed procedures of analysis are described in Materials and Methods. Briefly, AIH-related targets were collected from DisGeNET and GeneCards. The potential targets of costunolide were obtained from SwissTargetPrediction and PharmMapper. The potential targets of costunolide in AIH were generated by taking intersection of targets above. KEGG and GO enrichment analysis were performed using DAVID and Metascape. STRING database and Cytoscape were used for the construction of PPI network analysis. The docking between costunolide and key targets were conducted by PyMol and AutoDock. In vivo experiments were performed to verify the effect of costunolide on AIH and its underlying mechanism.

Network pharmacology was proposed by integrating network biology and polypharmacology [16]. It is capable of describing complexities among biological systems, drugs, and diseases from a network perspective, which has been applied in many studies to explore the molecular mechanism of drugs [17,18,19,20,21]. In this study, we investigated the effect of costunolide in two murine models of AIH: Concanavalin A (ConA)-induced acute immune-mediated hepatitis and human Cytochrome P4502D6 (CYP2D6) plasmid injection-induced chronic autoimmune hepatitis [22]. In addition, we integrated network pharmacology with experimental validation to clarify underlying mechanisms. Our research indicates that costunolide might be a potential option for the therapy of AIH. The flow chart of this research is shown in Figure 1. 

## 2. Results

### 2.1. Identification of the Potential Targets of Costunolide in AIH

**Figure 2:**
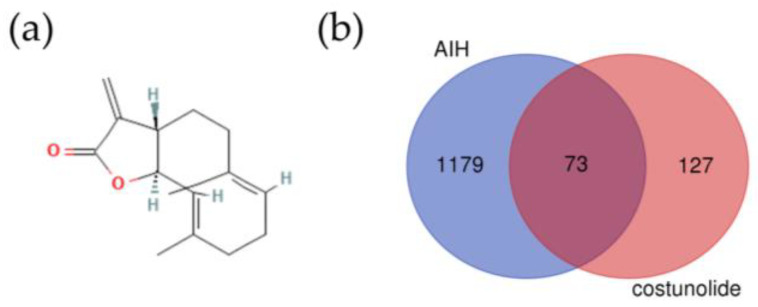
Potential targets of costunolide in AIH: (**a**) 2D structure of costunolide (PubChem Identifier: CID 5281437 https://pubchem.ncbi.nlm.nih.gov/compound/5281437#section=2D-Structure (accessed on 26 February 2022)), (**b**) Venn diagram of intersecting targets of costunolide and AIH.

Costunolide is a lactone compound isolated from Aucklandiae Radix. The two-dimensional (2D) structure of costunolide is shown in Figure 2a. The pharmacological and molecular properties of costunolide are shown in Table 1. Costunolide shows good oral bioavailability (OB) and high drug likeness (DL) in both the Traditional Chinese Medicine Systems Pharmacology Database and Analysis Platform (TCMSP) and SwissADME. 

From SwissTargetPrediction, we got 33 potential targets, and we retrieved 174 targets from PharmMapper. After merging data, removing duplicates, and confirming in UniProt, we got 200 potential targets of costunolide. Using the search word “autoimmune hepatitis”, we obtained 190 related genes in DisGeNET and 1183 genes which score >5 in GeneCards. After merging and removing duplicates, we obtained 1252 genes related to AIH. Via intersecting targets of costunolide and AIH-related genes, we obtained 73 potential targets of costunolide in AIH (Figure 2b). 

### 2.2. Recognition of Enriched Pathway of the Potential Targets of Costunolide in AIH

**Figure 3:**
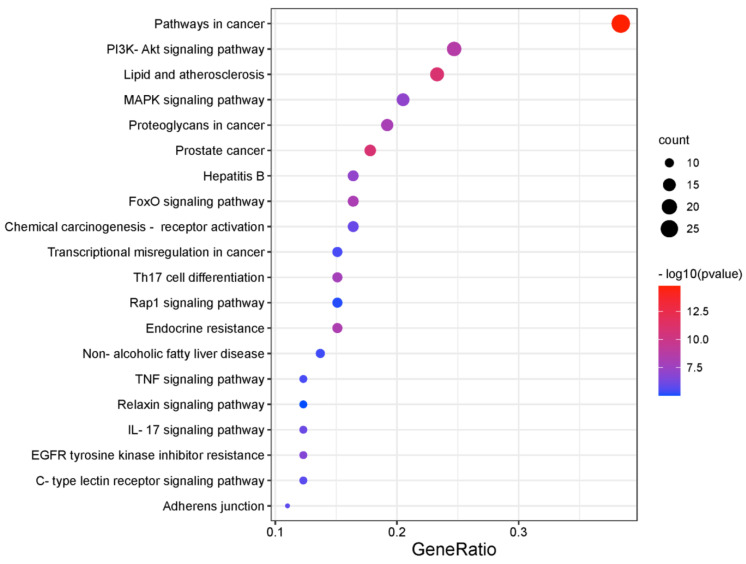
KEGG pathway enrichment analysis of intersecting targets of costunolide and AIH.

The Kyoto Encyclopedia of Genes and Genomes (KEGG) enrichment analysis was performed using The Database for Annotation, Visualization and Integrated Discovery (DAVID). The top 20 clusters were selected based on the *p*-value and were presented in the bubble chart (Figure 3). The potential targets of costunolide in AIH were involved in pathways in cancer (hsa05200), PI3K-AKT signaling pathway (hsa04151), lipid and atherosclerosis (hsa05417), MAPK signaling pathway (hsa04010), proteoglycans in cancer (hsa05205), etc. 

**Figure 4:**
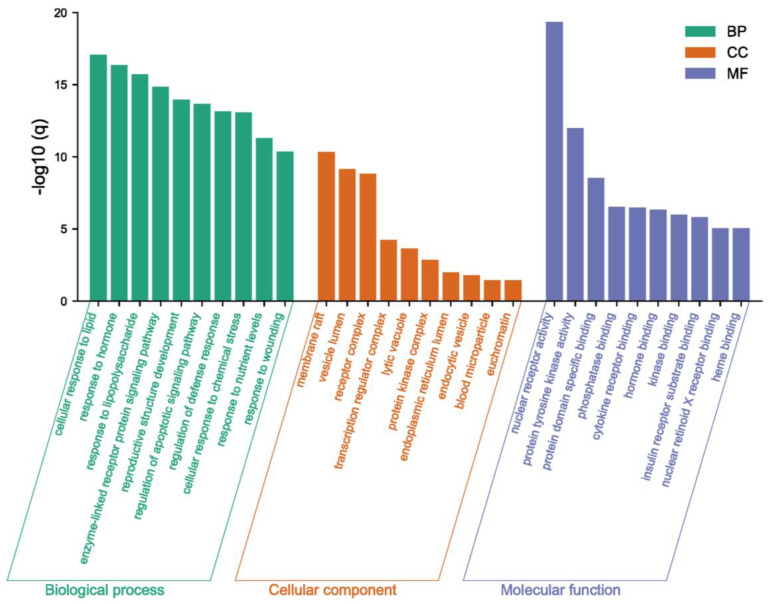
Gene Ontology (GO) enrichment analysis of intersecting targets of costunolide and AIH.

Meanwhile, based on the *p*-value of Gene Ontology (GO) enrichment analysis, the top 10 significantly enriched terms in Biological Process (BP), Cellular Component (CC), and Molecular Function (MF) categories were selected (Figure 4). Potential targets of costunolide in AIH were mainly involved in biological processes such as cellular response to lipid (GO:0071396), response to hormone (GO:0009725), and response to lipopolysaccharide (GO:0032496). The main cellular component terms were membrane raft (GO:0045121), vesicle lumen (GO:0031983), and receptor complex (GO:0043235). The main molecular function terms were nuclear receptor activity (GO:0004879), protein tyrosine kinase activity (GO:0004713), and protein domain specific binding (GO:0019904). 

### 2.3. Topological Network Analysis of the Potential Targets of Costunolide in AIH

**Figure 5:**
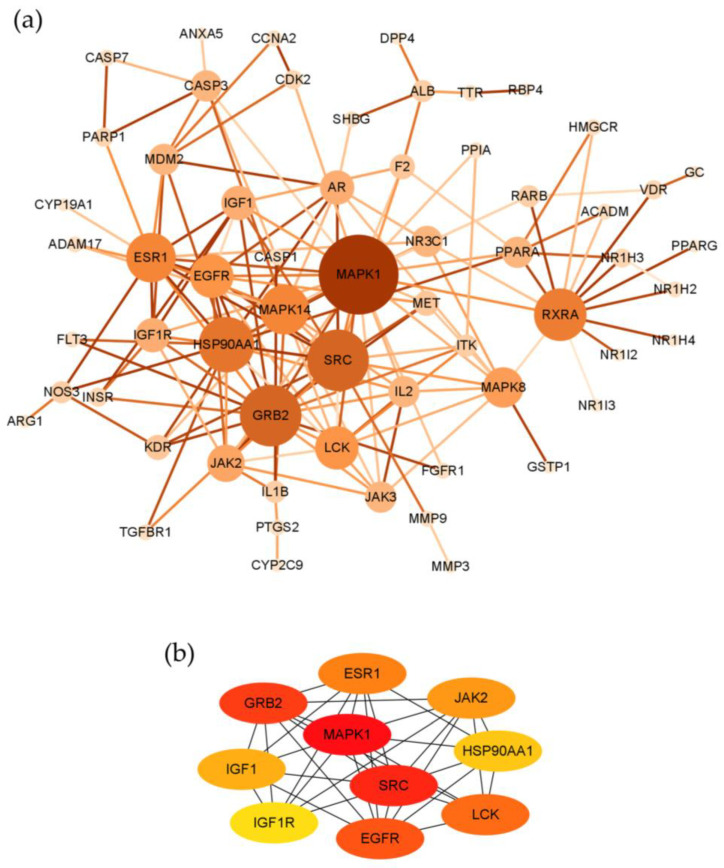
Protein–protein interaction network analysis. (**a**) Protein–protein interaction network of intersecting targets of costunolide and AIH visualized by Cytoscape. (**b**) The core subnetwork constructed by CytoHubba.

Seventy-three potential targets of costunolide in AIH were imported into The Search Tool of Retrieval of Interacting Genes (STRING) to construct the protein–protein interaction (PPI) network. After removing disconnected nodes, the network contained 61 nodes and 156 edges (Figure 5a). The average number of neighbors is 5.115 and the clustering coefficient is 0.317. Using plug-in CytoHubba, we found top 10 genes that might play critical roles in the therapeutic effect of costunolide in AIH, namely MAPK1, SRC, GRB2, EGFR, LCK, ESR1, JAK2, IGF1, HSP90AA1, and IGF1R, ranked by Maximal Clique Centrality (MCC) (Figure 5b). 

### 2.4. Molecular Docking

**Figure 6:**
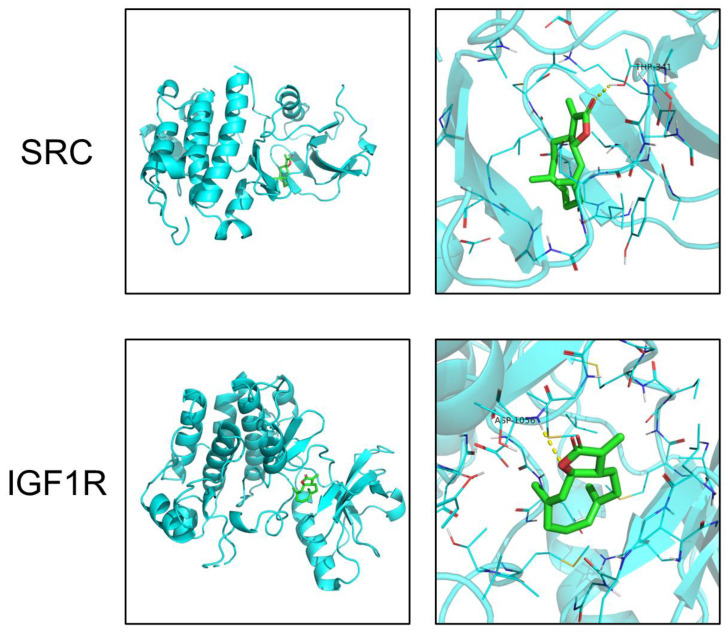
Molecular docking of costunolide with SRC and IGF1R.

Based on the pathway enrichment analysis and literature review, SRC and IGF1R ranked high in the PPI network analysis and were reported to regulate the PI3K-AKT pathway in inflammation-related diseases. However, their roles in AIH were not elucidated yet. Thus, we chose SRC and IGF1R as our interested targets of costunolide in AIH. Using PyMol and Autodock, molecular docking visually showed the interaction between costunolide and SRC, IGF1R. The diagrams of drug-target binding mode were shown left and the details right (Figure 6). The dotted yellow line represents the hydrogen bond. The lower binding energy indicates higher stability. The binding energies between costunolide and SRC and between costunolide and IGF1R were −7.7 and −5.9 kcal/mol, respectively (Table 2). 

### 2.5. Costunolide Attenuated ConA-Induced Acute Hepatitis

**Figure 7:**
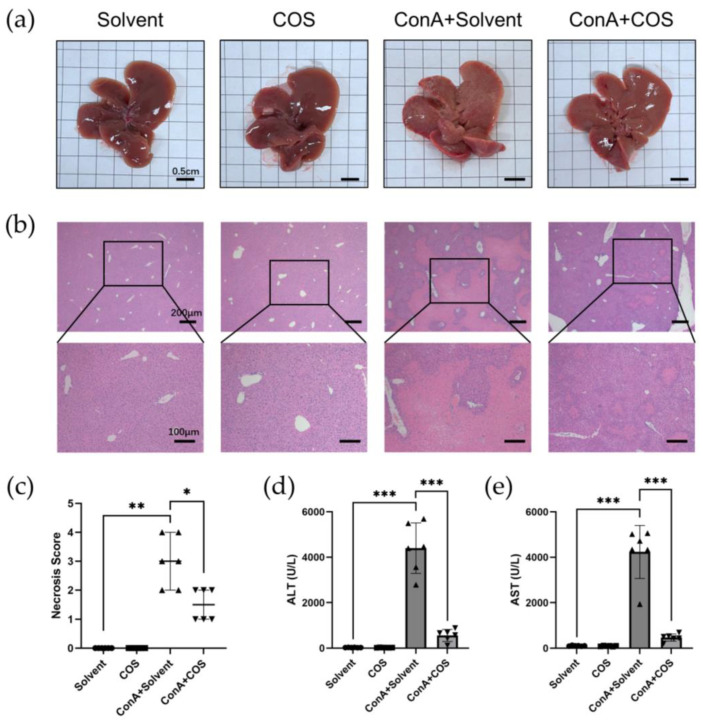
Costunolide attenuated ConA-induced acute hepatitis. (**a**) Liver gross appearance. (**b**) Representative H&E staining of liver sections. (**c**) Necrosis score of H&E staining of liver sections. (**d**) Effect of costunolide on serum ALT levels. (**e**) Effect of costunolide on serum AST levels. Data shown in c–e are from 6 individual mice per group. * *p* < 0.05, ** *p* < 0.01, *** *p* < 0.001.

Firstly, we administered costunolide in the ConA-induced acute hepatitis model. The liver gross appearance of ConA+Solvent mice showed hepatic congestion and suppuration on the surface, while ConA+COS liver showed a milder change (Figure 7a). In hematoxylin-eosin (H&E) staining, we observed that ConA induced necrosis of liver parenchyma. Costunolide administration significantly restrained the hepatic necrosis, while the necrosis score of ConA+Solvent and ConA+COS showed a significant difference (Figure 7b,c). Similar to the histological results, level of the liver enzyme alanine aminotransferase (ALT) and aspartate aminotransferase (AST) in serum surged in the ConA+Solvent group compared with Solvent and COS group, while costunolide significantly suppressed it (Figure 7d,e). Notably, there was no obvious difference of gross appearance, H&E staining, and liver enzyme level between the Solvent group and the COS group. 

### 2.6. Costunolide Ameliorated Chronic Murine Autoimmune Hepatitis

**Figure 8:**
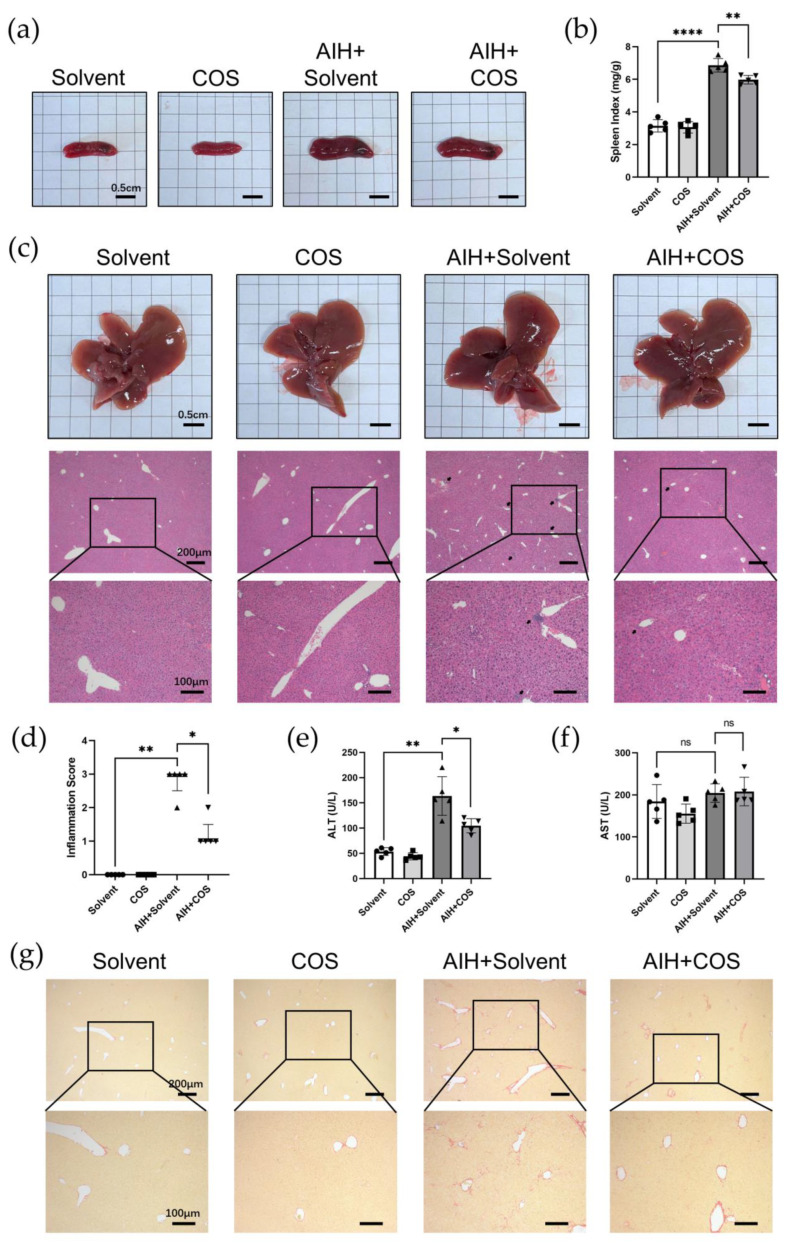
Costunolide ameliorated chronic murine autoimmune hepatitis. (**a**) Spleen gross appearance. (**b**) Spleen index (mg/g). (**c**) Representative liver gross appearance and H&E staining of liver sections (black arrows indicate immune cell infiltration). (**d**) Inflammation score of H&E staining of liver sections. (**e**) Effect of costunolide on serum ALT levels. (**f**) Effect of costunolide on serum ALT levels. (**g**) Representative Sirius-red staining of liver sections (Red color indicates collagen deposition). Data shown in (**b**,**d**,**e**) are from 5 individual mice per group. * *p* < 0.05, ** *p* < 0.01, **** *p* < 0.0001.

In our chronic murine AIH model, the spleens of AIH mice were enlarged significantly, while costunolide suppressed it (Figure 8a). There was a significant difference of spleen indexes between AIH+Solvent and AIH+COS mice (Figure 8b). Unlike ConA-induced acute hepatitis, there was no obvious change of the liver gross appearance in the chronic murine AIH model (Figure 8c). H&E staining of liver sections showed that costunolide inhibited immune cell infiltration in AIH mice, which is the typical characteristic of AIH (Figure 8c). There was a significant difference of inflammation score between AIH+Solvent and AIH+COS group (Figure 8d). The ALT level showed a similar trend as histological results (Figure 8e). There was no significant change of AST level in our chronic model (Figure 8f). Further, Sirius red staining of liver sections indicated that costunolide reduced liver fibrosis in the chronic AIH model (Figure 8g). 

**Figure 9:**
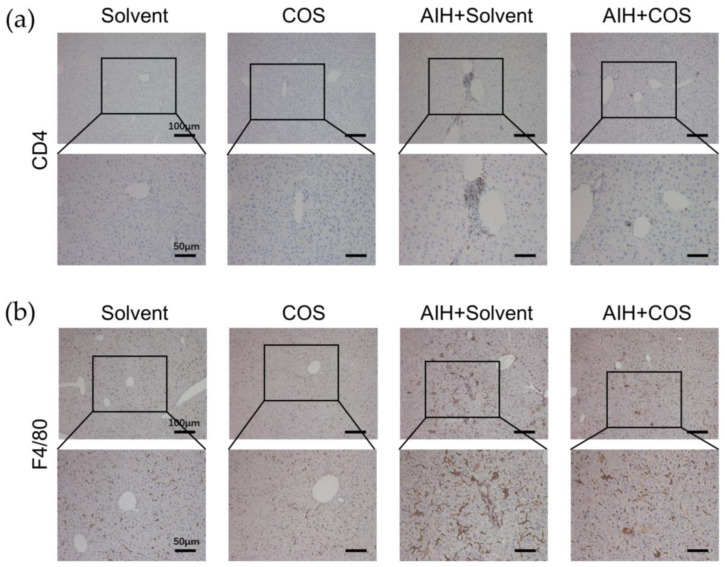
Costunolide inhibited immune cell infiltration in chronic murine autoimmune hepatitis. (**a**) Immunohistochemistry of CD4^+^ T cells. (**b**) Immunohistochemistry of F4/80^+^ macrophages.

To further elucidate the landscape of immune cell infiltration in different groups, we conducted immunohistochemistry (IHC) of CD4 and F4/80 in liver sections. The IHC result revealed the increased infiltration of CD4^+^ T cells and macrophages in AIH+Solvent mice, while costunolide suppressed this trend substantially (Figure 9a,b). 

### 2.7. Costunolide Inhibited the Activation of PI3K-AKT Pathway and Suppressed the Phosphorylation of SRC and IGF1R in AIH

**Figure 10:**
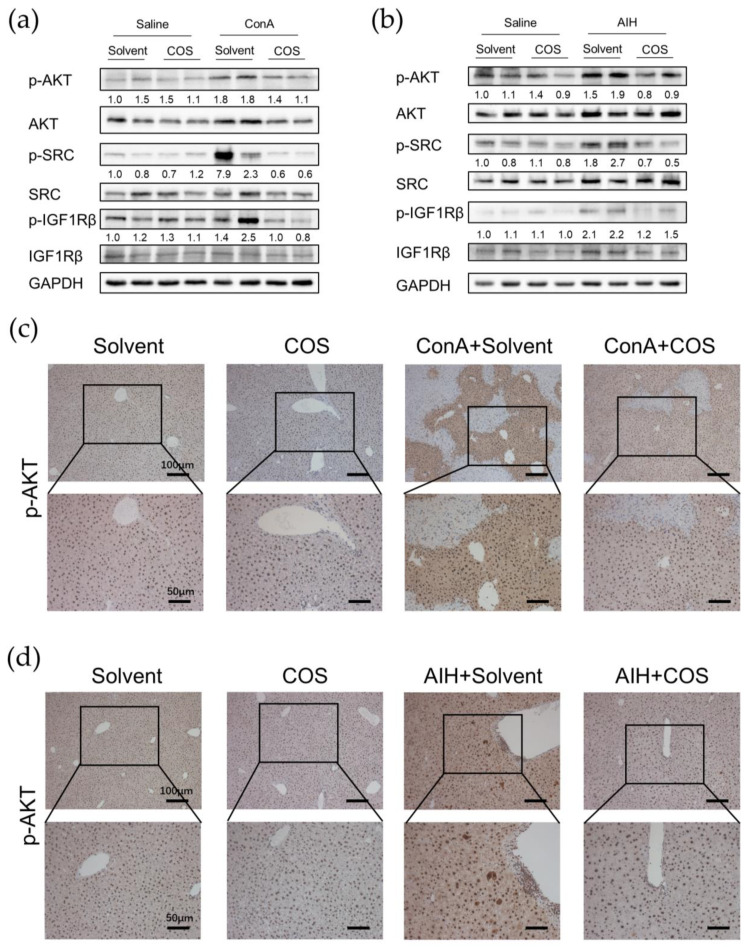
Costunolide inhibited the phosphorylation of AKT, SRC, and IGF1R. (**a**) Protein expression in ConA-induced acute hepatitis. Two samples from two different mice in each group are presented. The numbers indicate the ratio of pAKT/AKT, pSRC/SRC, and pIGF1Rβ/IGF1Rβ, respectively. (**b**) Protein expression in chronic murine autoimmune hepatitis. (**c**) Immunohistochemistry of phospho-AKT in ConA-induced acute hepatitis. (**d**) Immunohistochemistry of phospho-AKT in chronic murine autoimmune hepatitis.

Based on the pathway enrichment analysis, PPI network analysis and literature review, we investigated the protein level change of phosphorylated AKT, SRC, and IGF1R in liver tissue of two AIH models. The phosphorylation of AKT, SRC, and IGF1R were significantly increased in the ConA model, whilst costunolide dramatically inhibited it (Figure 10a, Supplementary Figure S1a). Similar results were found in the chronic AIH model (Figure 10b, Supplementary Figure S1b).

To further confirm this trend, we conducted IHC of phospho-AKT in liver sections. The phosphorylated AKT was mainly expressed in nuclei of hepatocytes in Solvent and COS mice, and its expression level was higher in two murine AIH models, mostly in hepatocytes nuclei and cytoplasm as well as infiltrating immune cells. The phospho-AKT expression level in ConA/AIH+COS groups were lower than that in the ConA/AIH+Solvent groups (Figure 10c,d).

## 3. Discussion

Autoimmune hepatitis (AIH) is a chronic liver disease related to immunological tolerance disorders targeting hepatocytes. Although AIH has long been considered a rare disease, several studies have indicated that the incidence and prevalence of AIH were increasing worldwide [25]. Although most patients respond well to traditional immunosuppressive therapy, many patients were troubled by the intolerance, insufficient response to medication, and recurrence after withdrawal of treatment [2]. The discovery of novel therapeutic strategies is necessary for the management of the increasing population of AIH. Costunolide is a promising candidate due to its anti-inflammatory and hepatoprotective function in other disease models, whereas there is no study about its effect in AIH yet [6,12,14]. In this study, we integrated network pharmacology and experimental validation to elucidate the therapeutic effect and underlying mechanism of costunolide against AIH. 

In the current study, we identified 73 targets that might participate in the effect of costunolide on AIH. Based on the KEGG enrichment pathway analysis and literature review, we speculated that costunolide might mainly restrain the disease progress of AIH through PI3K-AKT pathway. Generally, the PI3K-AKT pathway is an important intracellular signaling pathway for cell cycle progression, cell survival, and metabolism [26]. It is reported that IHC of liver sections from AIH patients showed upregulation of phosphorylated AKT [27]. In ConA-induced hepatitis, PI3K-AKT pathway is activated in hepatocytes and dendritic cells, promoting inflammation via inducing the transcription of IL-6 and activation of CD8^+^ T cell responses, respectively [28,29]. Similarly, in our experiments, we observed a significant elevated protein level of phosphorylated AKT in the liver of ConA-induced hepatitis and chronic AIH model in WB, and costunolide administration induced a decrease of it. This finding was further confirmed by IHC showing a similar trend as in WB, while the upregulated phosphorylated AKT in AIH models was mainly expressed in hepatocytes and infiltrating immune cells. In hepatocytes, the activation of AKT generally plays a protective effect [30]. However, it was recently reported that AKT activation in hepatocytes was also associated with increased inflammatory cytokine expression [28]. Activation of the PI3K-AKT-mTOR pathway and PI3K-AKT-FOXO1 signaling axis regulates the activation, differentiation, and function of T cells in different conditions [31,32,33,34,35]. Attenuation of PI3K signaling could lead to defects in T cell activation and neutrophil migration, restraining the inflammation in systemic lupus erythematosus (SLE) and rheumatoid arthritis (RA) models [36,37]. Several studies have reported the suppressing effect of costunolide on PI3K-AKT pathway in cancer cells and in DSS-induced colitis [11,26,38]. These findings suggest that the therapeutic effect of costunolide against AIH might be related to the inhibition of PI3K-AKT pathway, whereas the specific cell types should be studied further. Contrary to our results, it was reported that phospho-AKT was decreased in liver of ConA-induced hepatitis and in ConA-treated L02 cells compared with the saline control. This difference might be due to the different dosages of ConA and the time between ConA injection and sacrifice. Moreover, directly administrating ConA on L02 cells might not reflect the disease feature of immune-mediated hepatocyte destruction in vivo [39]. Interestingly, the top three signaling pathways of KEGG analysis included lipid and atherosclerosis pathway. It is reported that pre-existing high-fat diet-induced non-alcoholic fatty liver disease (NAFLD) in mice potentiates the severity of AIH [40]. Whether the lipid metabolism is involved in the pathophysiology of AIH and if costunolide could interfere with it awaits further research. 

Based on the PPI network analysis using STRING and CytoHubba, we further identified 10 important targets in the therapeutic effect of costunolide in AIH, namely MAPK1, SRC, GRB2, EGFR, LCK, ESR1, JAK2, IGF1, HSP90AA1, and IGF1R, ranked by MCC. After comprehensive consideration of KEGG pathway analysis, GO enrichment analysis, molecular docking, and literature review, we selected SRC and IGF1R as our candidate targets for further research. SRC belongs to the non-receptor protein-tyrosine kinases family, playing key roles in cell growth, division, migration, and survival signaling pathways [41]. Multiple studies indicated that SRC regulated the PI3K-AKT pathway in inflammation-related diseases such as multiple sclerosis (MS), Grave’s Disease (GD), and in the neutrophil extracellular traps (NET) formation [42,43,44]. In our experiments, we found that the phosphorylation level of SRC was significantly upregulated in hepatic tissue in two AIH models and was drastically decreased in the costunolide group. Similar to the present literature, our results provided clues that SRC phosphorylation rose in AIH, and it might exacerbate AIH via the PI3K-AKT pathway, which demands further rigorous research to confirm it. Moreover, another protein in SRC family, LCK, also ranked high in our network analysis and it is an important regulator in T cell proliferation and function [45]. Whether costunolide also interacts with LCK in its effect against AIH is an interesting focus for further studies. Insulin-like growth factor-1 receptor (IGF1R), the primary signaling receptor of insulin-like growth factors (IGF) system, signals through the PI3K-AKT-mTOR and RAS-RAF-MEK-ERK pathways [46]. Dysregulation of the IGF system has been directly related to altered CD4^+^ T cell function in RA and GD [47,48]. Although there is no report about the direct contributory role of IGF1R in AIH yet, recently, its specific modulatory role in autoimmunity was confirmed by researchers in EAE mice. It augmented AKT-mTOR and STAT3 signaling, favoring Th17 cell differentiation over that of Treg cells [46]. Our results showed that phosphorylated IGF1R level was increased in the liver of AIH models, and its level decreased in the costunolide group, indicating that IGF1R phosphorylation level was altered in AIH, and costunolide might attenuate AIH via inhibiting the phosphorylation of IGF1R. Since the appropriate balance of Th17 and Treg cells maintains immune tolerance and impairment of it permits progress of AIH, the variation of IGF1R might be related to AIH progression by modulating Th17/Treg balance by regulating the PI3K-AKT pathway, which should be studied further. 

In this study, we found costunolide possessed a strong therapeutic effect in two AIH models. Transaminase is the sensitive indicator of the liver injury. In our research, we observed that the administration of costunolide could lower the elevated transaminase level in two murine AIH models. The change of AST level was not statistically significant in our chronic AIH model, which might be due to the relatively overall lower degree of liver inflammation. As is shown in H&E staining, the necrosis area was also diminished by costunolide in the ConA model. The spleen index (spleen weight/ body weight) could roughly reflect the degree of chronic inflammation [49]. We found that the spleen index in AIH group was significantly higher than the Solvent group and COS group, while the spleen index of COS+AIH group was significantly lower, indicating that costunolide could suppress the chronic inflammation. This finding was further supported by the suppressed immune cell infiltration in liver observed in H&E and the reduced CD4^+^ T cells and macrophage infiltration in liver as is shown in IHC. Based on the present literature, costunolide inhibits the production of pro-inflammatory factors in macrophages and influences the differentiation of CD4^+^ T cells in vitro [50,51]. Since T cells and macrophages both play pivotal roles in AIH, the specific mechanism of costunolide attenuating AIH and the exact role of PI3K-AKT pathway inside await further investigation such as flow cytometry. We also observed that costunolide suppressed liver fibrosis in chronic AIH model. That antifibrotic function might mainly be due to its inhibition of inflammation, which removed the etiological factor causing liver injury [52]. It also could be related to its direct antifibrotic effect via inhibiting hepatic stellate cell (HSC) activation as reported before [15]. It is worth noting that methacrylic acid copolymer (MAC)-coated PH-responsive mesoporous silica nanoparticles (MSNs) carrying costunolide significantly repressed liver fibrogenesis at a reduced dose in vitro and in vivo [53]. Adopting similar technology might also reduce the dosage needed for the therapeutic effect of costunolide against AIH. 

This study has some limitations. The binding affinity of costunolide with potential targets awaits further verification using surface plasmon resonance (SPR) or bio-layer interferometry (BLI) assays. The specific cell types in which those phosphorylated protein level increased are not identified. Since in different cell types those signaling pathways might exert distinct roles, we plan to perform immunofluorescence to reveal it. The specific molecular mechanism is not clear due to no rescue experiment being conducted. In addition, the CYP2D6 model reflects type 2 AIH. The conclusion of our study remains to be confirmed in more AIH models. 

## 4. Materials and Methods

### 4.1. Chemical Information Collection and Targets Retrieval of Costunolide 

The molecular and pharmacological properties data of costunolide were obtained from TCMSP [23] and SwissADME [24]. The canonical SMILES (Simplified Molecular-Input Line-Entry System), image, and SDF format file of 2D structure of costunolide were obtained from the PubChem database (PubChem Identifier: CID 5281437 https://pubchem.ncbi.nlm.nih.gov/compound/5281437#section=2D-Structure (accessed on 26 February 2022)) [54]. SwissTargetPrediction [55] and PharmMapper [56] were utilized to predict the potential targets of costunolide. For outcome of PharmMapper, targets which norm fit score > 0.25 were included in subsequent analysis. After integrating data and removing duplicates, all proteins were verified in the UniProt database [57].

### 4.2. Prediction of Potential Targets of Costunolide in AIH and Enrichment Analysis

AIH-related targets were collected from DisGeNET [58] and GeneCards (https://www.genecards.org/ (accessed on 21 June 2022)) [59]. In GeneCards, genes that Score > 5 were included in subsequent analysis. The potential targets of costunolide and AIH-related targets obtained in above steps were imported into an online Venn diagram drawing tool (https://bioinformatics.psb.ugent.be/webtools/Venn/ (accessed on 13 September 2022)). 

KEGG enrichment analysis was conducted using DAVID [60,61]. Species was set as *Homo sapiens*. Threshold and EASE were set as 2 and 0.1, respectively. GO enrichment analysis was performed using Metascape [62]. Species was set as *Homo sapiens* and all genes in the genome were used as the enrichment background. Min overlap, *p* value cutoff, and min enrichment were set as 3, 0.01, and 1.5, respectively. Bubble chart and bar graph were plotted by http://www.bioinformatics.com.cn (accessed on 14 September 2022), an online platform for data analysis and visualization. 

### 4.3. Protein–Protein Interaction Target Network Construction and Visualization

STRING database (https://cn.string-db.org/ (accessed on 14 September 2022)) was utilized to construct a PPI network [63]. The organism was set as *Homo sapiens*. The minimum required interaction score was set as 0.900 (highest confidence), and disconnected nodes in the network were hidden. 

Network visualization and analysis were performed using Cytoscape version 3.9.1 [64]. The TSV-format file was downloaded from the STRING database and imported into Cytoscape. The size and color of nodes were defined continuously according to the degree (number of edges), and the color of stroke was set according to the combined score calculated by network analyzer of Cytoscape. The plug-in CytoHubba (https://apps.cytoscape.org/apps/cytohubba (accessed on 14 September 2022)) was used to find the core subnetwork. The node’s scores were calculated and top 10 hub genes ranked by MCC were selected, then the network graph was constructed. 

### 4.4. Molecular Docking

The crystal structures of candidate targets were obtained from the PDB database (https://www.rcsb.org/ (accessed on 20 September 2022)). Open-Source PyMol was used to pre-process the structure, including removing the ligand and water molecules, adding hydrogen [65]. AutoDock 4.2 was used to conduct docking between costunolide and key targets [66]. 

### 4.5. Establishment of Two Experimental AIH Models and Administration of Costunolide

C57BL/6 mice were supplied by the Laboratory Animal Centre of Tongji Hospital. All animals were housed in the SPF environment and a 12 h light/12 h dark cycle at room temperature of 22 ± 2 °C with 55 ± 2% humidity. All animals had free access to food and water. The animal study protocol was approved by the Laboratory Animal Welfare and Ethics Committee of Tongji Hospital of Tongji Medical College, Huazhong University of Science and Technology (IACUC Issue No.: TJH-202207036; date of approval: 15 July 2022). The specific animal model establishment procedure and treatment scheme was depicted in supplementary materials (Supplementary Figure S2). 

Concanavalin A (ConA) was used to establish the acute immune-mediated liver injury model. ConA (C2010, CAS: 11028-71-0, LOT: 091610120V) was purchased from Sigma-Aldrich. Costunolide (CAS: #553-21-9, LOT: J22GB152180) was purchased from Shanghai YuanYe Bio-Technology Co., Ltd. Twenty-four male mice (6–8 weeks old, 20–25 g) were randomly divided into solvent group (Solvent, *n* = 6), costunolide group (COS, *n* = 6), ConA group (ConA+Solvent, n = 6), and ConA+costunolide group (ConA+COS, *n* = 6). A single dose of ConA (15 mg/kg) dissolved in normal saline was injected via tail vein of mice, and the same volume of normal saline injection was used as control. Based on the existing literature, costunolide (10 mg/kg) was injected intraperitoneally once daily for three days, the first dose was given two days before giving ConA [11,26]. Costunolide was dissolved in DMSO, then mixed with 40% PEG300 (CAS: 25322-68-3, MCE), 5% Tween 80 (CAS: 9005-65-6, MCE), and normal saline. Mice were sacrificed 24 h after ConA injection. After anesthesia, blood samples were collected by removing the eyeball from the socket using tissue forceps [67]. Mice were then sacrificed and livers were harvested. Part of the liver was fixed in 4% paraformaldehyde for 48 h, the rest was collected in EP tubes, cooled in liquid nitrogen, and preserved in a −80 °C refrigerator. 

As described in our previous publication, a single dose of empty adenovirus and multiple high-pressure tail vein injections of human CYP2D6 plasmid were administered to establish the chronic AIH model [22]. Empty adenovirus was purchased from Shanghai DesignGene Company. Human CYP2D6 plasmid was obtained from our lab. Twenty male mice (6–8 weeks old, 20–25 g) were randomly divided into four groups: solvent group (Solvent, *n* = 5), costunolide group (COS, *n* = 5), AIH group (AIH+Solvent, *n* = 5), and AIH+costunolide group (AIH+COS, *n* = 5). In the COS group and AIH+COS group, costunolide (10 mg/kg) was injected intraperitoneally once daily from day 14 after the adenovirus injection continuously for 20 days. Mice were sacrificed at day 34 after adenovirus injection. The sacrifice and specimen taking procedure was similar to the ConA model described above. In addition, spleens were harvested to measure the spleen weight, and spleen index was calculated using spleen weight (mg) divided by mice weight (g). 

### 4.6. Histopathology, Immunohistochemistry, and Biochemical Analysis

The fixed liver tissue was embedded in paraffine and sliced at a thickness of 4 µm. H&E staining was performed to evaluate liver injury and inflammation. Afterward, all sections were graded of whole sections blindly under optical microscope (Olympus IX71) and representative pictures were imaged. Necrosis scores and inflammation scores were calculated as below: 0, no area of necrosis/immune cells infiltration; 1, very mild, few interspersed necrosis/ infiltration; 2, mild, necrosis/infiltration area ≤30%; 3, moderate, 30% < necrosis/infiltration area ≤60%; 4, severe, necrosis/infiltration area >60%. Plus, Sirius-red staining was performed to evaluate the degree of liver fibrosis in chronic AIH model. Immunohistochemistry (IHC) was performed to detect the expression of CD4, F4/80, and phospho-AKT expression in liver tissue according to protocol using SP9000 IHC kit (ZSGB-Bio, Beijing, China). Primary antibodies: anti-CD4 (1:2000; ab183685, Abcam (Cambridge, UK)), anti-F4/80 (1:200; #70076S, CST (Danvers, MA, USA)), and anti-phospho-AKT (Ser473) (1:250; AF0016, Affinity (Cincinnati, OH, USA)) were used. 

Blood samples collected were centrifuged at 3000 rpm for 15 min to obtain the serum. The liver enzyme ALT and AST in serum were measured using the Rayto automatic biochemistry analyzer Chemray 420 by Wuhan ServiceBio Company (Wuhan, China).

### 4.7. Western Blot

Liver tissue specimens were preserved in a −80 °C refrigerator before use. After being cut to an appropriate size, liver tissue was lysed in RIPA lysis buffer (harsh) containing protease inhibitor cocktail and phosphatase inhibitor cocktail (Wuhan Servicebio Technology Co., Ltd. (Wuhan, China)). Homogenizer was used to grind tissue into homogenate and sonication was performed using ultrasonic processor. After 40 min lysis, all samples were centrifuged at 12,000 rpm for 10 min. Protein concentration was measured using BCA kit (Wuhan Servicebio Technology Co., Ltd. (Wuhan, China)). Tissue protein (40 µg) was separated in an SDS-PAGE and was transferred to PVDF membranes. The blotted PVDF membranes were blocked with TBST containing 5% BSA at room temperature for 1 h. Then the primary antibodies were incubated overnight at 4 °C including: anti-phospho-AKT (Ser473) (1:1000; #4060T, CST), anti-AKT(1:1000; #9272S, CST), anti-phospho-SRC (Tyr416) (1:1000; #6943S, CST), anti-SRC (1:1000; #A19119, Abclonal (Wuhan, China)), anti-phospho-IGF1Rβ (Tyr1135/1136) (1:1000; #3024S, CST), anti-IGF1Rβ (1:800; #9750S, CST), anti-GAPDH (1:2000; 30202ES60, Yeason (Shanghai, China)). On the next day, secondary antibodies were incubated for 1 h at room temperature. After being washed 3 times in TBST for 10 min, membranes were exposed to hypersensitive electrochemiluminescence (ECL) kit (NCM Biotech (Suzhou, China)). Protein bands were visualized on the Tanon 5200 Multi System (Tanon Science and Technology Co., Ltd. (Shanghai, China)).

### 4.8. Statistical Analysis

GraphPad Prism software (version 9.0.0) was utilized to analyze data. The continuous data was presented as mean ± standard deviation (SD), and the Student’s *t*-test was performed to analyze the comparison between two groups. Welch’s correction was used if variances were not equal based on the F test. The discontinuous data were analyzed using the Mann–Whitney test. *p* < 0.05 was considered statistically significant. 

## 5. Conclusions

In our present study, network pharmacology and experimental validation were integrated to investigate the effect and mechanism of costunolide in AIH. According to the results, costunolide could relieve the inflammation and fibrosis in two murine AIH models, and the therapeutic effect might work by suppressing the activation of PI3K-AKT pathway and inhibiting the phosphorylation of SRC and IGF1R. Our research reveals the effect and possible mechanism of costunolide in AIH and may further contribute to novel drug development for AIH and other autoimmune diseases. 

## Figures and Tables

**Table 1:** Pharmacological and molecular properties of costunolide.

MW	AlogP	Hdon	Hacc	OB (%)	Caco-2 (nm/s)	BBB	DL	FASA-	TPSA	RBN
232.35	4.02	0	2	29.07	1.28	1.42	0.11	0.36	26.30	0

Abbreviations: MW, molecular weight; AlogP, low lipid/water partition coefficient; Hdon, hydrogen bond donors; Hacc, hydrogen bond acceptors; OB, oral bioavailability; Caco-2, Caco-2 permeability; BBB, blood brain barrier; DL, drug likeness; FASA-, fractional water accessible surface area of all atoms with negative partial charge; TPSA, topological polar surface area; RBN, rotatable bonds number. The molecular and pharmacological properties data of costunolide were obtained from TCMSP [23] and SwissADME [24].

**Table 2:** Binding energies of costunolide with SRC and IGF1R.

Target	PDB ID	Binding Energy (kcal/mol)
SRC	6E6E	−7.7
IGF1R	3NW7	−5.9

## Data Availability

All data generated or analyzed during this study are included in this article and the supplementary material.

## Supplementary Materials

The following supporting information can be downloaded at: https://www.mdpi.com/article/10.3390/ph16020316/s1, Figure S1: Costunolide inhibited the phosphorylation of AKT, SRC, and IGF1R. Figure S2: Animal model and experimental design.
