# Supplementary material for: Therapeutic Effect of Costunolide in Autoimmune Hepatitis: Network Pharmacology and Experimental Validation
Source: Pharmaceuticals (Basel). 2023 Feb 17;16(2):316. doi: 10.3390/ph16020316 (PMC9963495; doi:10.3390/ph16020316)
Supplement: Supplementary file 1 [file pharmaceuticals-16-00316-s001.zip › pharmaceuticals-2197005-supplementary.pdf]

Supplementary Figures

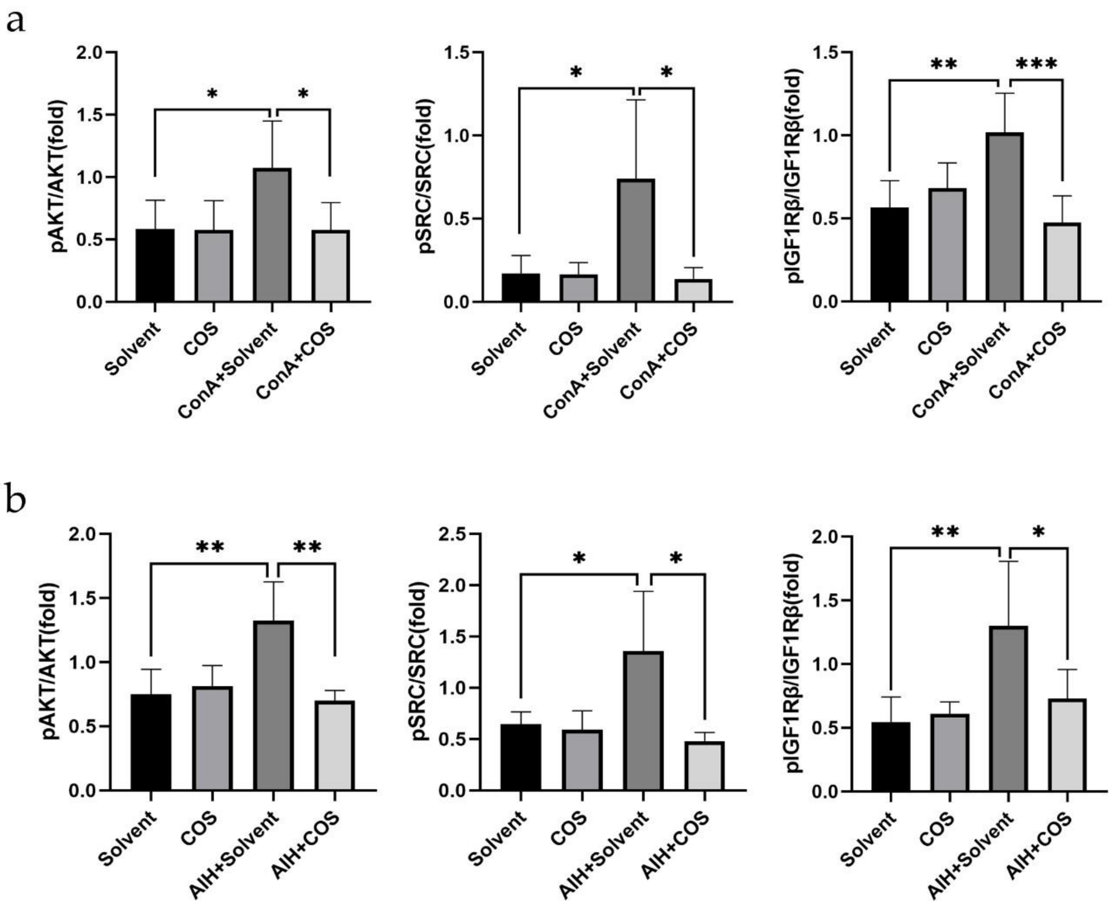

Supplementary Figure S1. (a) Protein expression fold in ConA-induced acute hepatitis. (b) Protein expression fold in chronic murine autoimmune hepatitis. * $p < 0.05$, ** $p < 0.01$, *** $p < 0.001$.

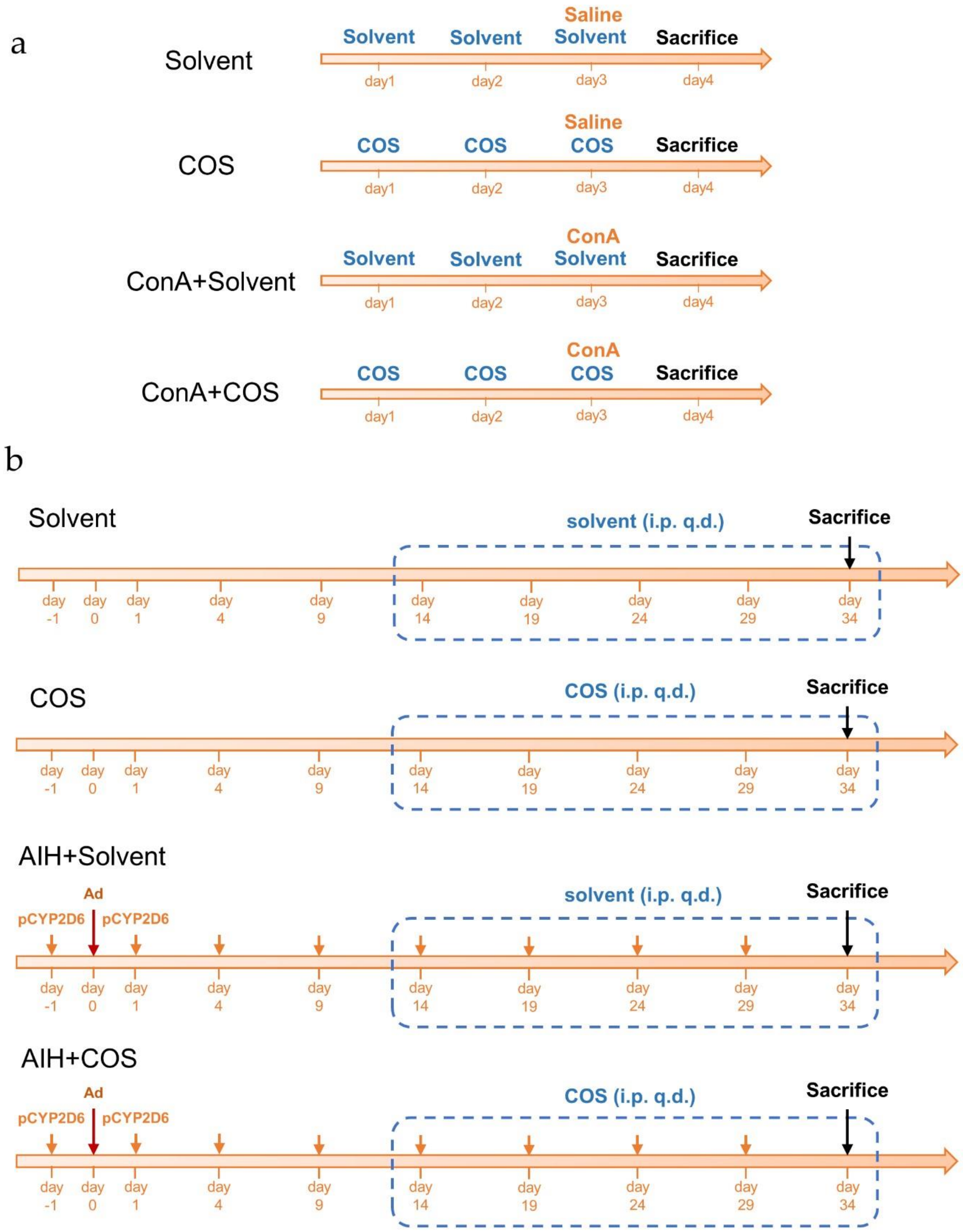

Supplementary Figure S2. Animal models and experimental design. (a) The ConA-induced acute hepatitis model. (b) The chronic murine autoimmune hepatitis model.
